# Seroprevalence, risk factors and impact of *Toxoplasma gondii* infection on haematological parameters in the Ashanti region of Ghana: a cross-sectional study

**DOI:** 10.12688/aasopenres.13022.2

**Published:** 2020-06-17

**Authors:** Samuel Kekeli Agordzo, Kingsley Badu, Mathew Glover Addo, Christian Kwasi Owusu, Abdul-Hakim Mutala, Austine Tweneboah, Dawood Ackom Abbas, Nana Kwame Ayisi-Boateng

**Affiliations:** 1 Theoretical and Applied Biology, Kwame Nkrumah University of Science and Technology, Kumasi, AK 192, Ghana; 2 Kumasi Centre for Collaborative Research for Tropical Medicine, Kwame Nkrumah University of Science and Technology, Kumasi, Ghana; 3 School of Medicine and Dentistry, Kwame Nkrumah University of Science and Technology, Kumasi, Ghana; 4 The University Hospital, Kwame Nkrumah University of Science and Technology, Kumasi, Ghana

**Keywords:** Toxoplasma gondii, Haematology, Seroprevalence, IgG, Exposure, Risk Factors

## Abstract

**Background: **Toxoplasma gondii is an obligate, intracellular, apicomplexan parasite that causes toxoplasmosis. Although the global prevalence of toxoplasmosis has been estimated to be approximately 30%, there is limited seroprevalence data in Ghana, with a dearth of information on the impact of T. gondii on haematological parameters in exposed persons.

**Methods: **Questionnaires were administered to 300 consenting individuals to obtain demographic information and assessment of their risk of exposure to *T. gondii*. Using anti- *T. gondii *IgG/IgM combo test kits, seropositivity to parasite-specific IgG and/or IgM was determined. A haematological analyser was used to measure haematological parameters.

**Results: **There was an overall seroprevalence of 50.3% (n=151), with 49.7% (n=149) of the study participants seropositive for IgG and 1% (n=3) testing positive for IgM. Furthermore, the observed seroprevalence among pregnant women was 56.4% (n=62). With regard to settlement type, a seroprevalence of 55.6% was observed in the rural community, 50.6% in the peri-urban community and 47.1% in the urban community. The study identified cat ownership, contact with cat litter, contact with raw meat  [RR (95% CI: 1.76 (1.23-2.53), 1.66 (1.03-2.67), 1.25(1.00-1.57)] and age (p<0.001) as risk factors for infection. Analyses of haematological data revealed significant reduction in the white blood cell, lymphocytes and mean corpuscular volume levels in seropositive males (p=0.0223, 0.0275, and 0.0271) respectively. Only the mean corpuscular volume of seropositive females reduced significantly as compared to the seronegative counterparts (p=0.0035).

**Conclusions:** About half of the study population, including women of reproductive age carried antibodies against *T. gondii*, raising concerns about the risk of congenital toxoplasmosis and anaemia. We, therefore, recommend that screening for *Toxoplasma gondii *be included in the routine screening of pregnant women seeking antenatal care and further investigation should be conducted on the haematological implications of infection in humans.

## Introduction

*Toxoplasma gondii* — the causative organism for toxoplasmosis — is an obligate, intracellular, apicomplexan parasite with a wide geographic distribution and the ability to infect virtually any cell type across a broad host range, including humans, companion animals, livestock and wildlife ^1^. About a third of the world’s population is infected with *T. gondii* but the parasite does not usually cause clinically significant disease ^2^. Until recently, latent infections in humans were assumed to be asymptomatic; however, results from animal studies, personality and behavioural profiles, as well as psychomotor performance tests have led to a reconsideration of this assumption ^3, 4^. Certain individuals, including foetuses, neonates, and the immune-compromised are at high risk for life-threatening complications from toxoplasmosis ^4^. Congenital transmission of *T. gondii* carries the risk of miscarriage or stillbirth, and children born with toxoplasmosis are likely to suffer from severe symptoms, such as hydrocephalus, calcifications of the brain or retinochoroiditis later in life if not treated ^5^.

People are typically infected by either accidentally ingesting infectious oocysts or eating undercooked meat containing tissue cysts (bradyzoites). Toxoplasma oocysts can be found in soil or water contaminated with cat faeces, making the consumption of raw vegetables and water from unsafe drinking sources important risk factors for infection. The consumption of raw or undercooked meat is also a risk factor as livestock and game often harbour bradyzoites. The parasite can also be transmitted *in utero* to a developing foetus if a woman is infected for the first time while pregnant. Solid-organ transplantation such as the heart, liver and kidney transplants are another means by which *T. gondii* infection can occur, although this is very rare ^6, 7^.

During acute primary infection with *T. gondii*, anti- *T. gondii* immunoglobulin M (IgM) is initially produced. However, IgM titres decline over the next few months, becoming undetectable within a year. The immune system also produces anti- *T. gondii* IgG a few weeks after the initial infection. IgG antibody levels usually peak within one or two months after the infection but are still detectable throughout the lifetime of the infected individual ^4^. The seroprevalence of antibodies against *T. gondii* has been reported across the globe, and ranges from 51% to 72% in several countries in Latin America and the Caribbean, including Argentina, Brazil, Cuba, Jamaica, and Venezuela. In Scandinavia, the seroprevalence of antibodies specific to *T. gondii* is reported to vary between 11% and 28%, while in Southeast Asia, China and Korea, seropositivity to *T. gondii* exposure has been estimated to range from 4% to 39% in women of reproductive age ^8^. Furthermore, both active and latent infections of *T. gondii* have been reported in many African countries, particularly in individuals suffering from HIV/AIDS. For example, 88.2% of HIV-positive individuals seeking healthcare at Arba Minch Hospital in Ethiopia were also seropositive for *T. gondii* infection ^9^, and a study conducted in Burkina Faso among pregnant women revealed an overall seroprevalence of 31.1% ^10^.

In Ghana, Sefa-Boakye *et al*., ^11^ and Ayi *et al*., ^12^ reported seroprevalence rates of 83.6% and 92.5% among pregnant women in Kumasi and Accra, respectively. In the Central region of Ghana, Abu *et al*., ^13^ reported an overall seroprevalence of 85% in a population-based study that investigated risk factors for *T. gondii* infection. A follow-up study later revealed that 2.6% of the study participants showed signs of ocular toxoplasmosis ^14^. In the Greater Accra region, Kwoffie *et al*., ^15^ analysed placental tissue samples from IgG-seropositive women post-delivery and estimated the risk of congenital transmission of the infection to be 39.8% based on the presence of *T. gondii* DNA in the placental samples.

In this study, we screened 300 individuals from three hospitals in the Ashanti region of Ghana and estimated the seroprevalence of *T. gondii* infection. Our primary objective was to investigate the seroprevalence, associated risk factors and haematological consequences of *T. gondii* infection in the Ashanti region of Ghana. Specifically, we determined 1) the seroprevalence of *T. gondii*-specific IgG and IgM; 2) the risk factors associated with *T. gondii* infection; and 3) the haematological consequences of *T. gondii* exposure in the Ashanti region of Ghana.

## Methods

### Ethical consideration

Prior to the commencement of the study, approval was obtained from all three hospitals. Ethical approval for the study was also given by the Committee on Human Research Publications and Ethics at the Kwame Nkrumah University of Science and Technology and Komfo Anokye Teaching Hospital in Kumasi, Ghana (reference number: CHRPE/AP/018/18). Written informed consent was obtained from all the study participants.

### Study design

A cross-sectional study design was employed. Study participants were recruited during hospital visits that occurred between 30 ^th^ January and 28 ^th^ February 2018. Questionnaires were administered to obtain data on demographics and risk factors.

### Study site

The study was conducted at three hospitals; namely, Kumasi South Hospital (KSH), Agona Government Hospital (AGH) and Kuntanase Government Hospital (KGH), all in the Ashanti region of Ghana. KSH is located in Atonsu, a community within the Asokwa Sub-metropolitan Assembly of the Kumasi Metropolis which has a population size of 1,730,249. Recruitment and sample collection were conducted from 31st January to 8th of February 2018 at KSH. At KGH, recruitment and data collection were carried out from 15 ^th^–16 ^th^ and 27 ^th^–28 ^th^ February 2018. KGH is situated in Kuntanase, the capital of the Bosumtwi district of the Ashanti Region with a population of 93,910 and located at about 28km from the capital city, Kumasi. AGH is located at about 37 km from Kumasi and is situated in the Sekyere South District, which has a population of 94,009. Recruitment and selection of participants took place from 19 ^th^–21 ^st^ February 2018 at the Agona Government Hospital.

### Participant selection

To be included in the study, individuals had to be at least six months of age and had to have been referred to the laboratories of one of the study hospitals (KSH, KGH and AGH). Pregnant women were preferentially recruited into the study due to our interest in congenital toxoplasmosis. The selection of study subjects was based on their willingness to participate after being briefed about the study, and confirmed by signing or thumb-printing an informed consent form. In the case of minors, consent was obtained from parents or legal guardians. Critically ill patients (individuals in need of urgent medical attention) and children below 6 months were excluded from the study. We provided a written questionnaire to each of the participants as they waited for their turn at the laboratory of the various hospitals. The questionnaires were administered in the form of an interview to all participants by the investigators.

### Variables

Data obtained from the questionnaires on factors that predispose respondents to infection (cat ownership, contact with cat litter, eating raw meat, consumption of raw or undercooked vegetables and sources of drinking water) were analysed. As part of the demographic data, age, sex, location (rural, urban or peri-urban), education and employment were also analysed. These variables were treated as predictor variables and compared with the anti- *T. gondii* serological results (endpoint variables).

### Bias

The female to male ratio was identified to be biased. This was due to priority being given to the enrolment of pregnant women.

### Sample size determination

A minimum sample size of 207 was determined using the binomial model. This was calculated with a 95% CI and precision level of 5%: N _o_= [Z ^2^ (P) (1-P)] /(d) ^2^ using overall seroprevalence of 83.4% observed by Sefah-Boakye *et al*. ^11^ in the Ashanti region. In this equation, N _o_ is the sample size, Z is the critical value of the binomial distribution at the 5% level (1.96), p is the overall seroprevalence (0.84), q = 1 – p, and d (error) is the precision level (5%).

### Quantitative variables

Participants who tested positive for parasite-specific IgG, IgM or both were classified as seropositive, while those with negative tests were described as seronegative. We compared the median haematological parameter values of the seropositive and seronegative categories to investigate the effects of infection on the different haematological indices.

### Haematological analyses

Using sterile needles and syringes, qualified phlebotomists obtained 3 mL of venous blood from each participant. The blood samples were dispensed into labelled EDTA tubes to prevent clotting and were later used for haematological analyses. Complete blood counts were run on the blood using the Sysmex XP-300 Automated Haematology Analyzer. The analyser provided cell count data on red blood cells (RBC), white blood cells (WBC), lymphocytes, neutrophils and platelets. Other parameters measured included haemoglobin (Hb), mean corpuscular volume (MCV), mean corpuscular haemoglobin (MCH), mean platelet volume (MPV), plateletcrit and red blood cell distribution width (RDW).

### Serological analyses

The blood samples were tested for the presence of anti- *T. gondii* IgG/IgM using the commercially available *OnSite* Toxo IgG/IgM rapid combo test kit manufactured by CTK Biotech, USA. This test kit works with either whole blood or serum. Following the manufacturer’s protocol, a 10 µL sample of blood was dropped into the sample/buffer well on the kit. Two drops of buffer were then added and allowed to stand for 10–15 min. Results were recorded as positive if both the test control line and M and/or G line(s) developed. Test results were seropositive for IgG if the G line developed in addition to the control (C) while blood samples were seropositive for IgM if the M line appeared in addition to the control. Seropositive results to IgG and IgM were obtained when both the M and G lines developed in addition to the control (C) band.

### Data analysis

Data obtained from serological examination and questionnaires were keyed into Microsoft Excel (2016). Seroprevalence was calculated by expressing the number of seropositive individuals as a percentage of the total number tested. The chi-square and Mann-Whitney U tests were carried out in GraphPad Prism 6 (GraphPad Software, Inc., San Diego, CA, USA) to investigate the association between demographics and seropositivity, as well as to compare the differences in the median haematological values of seropositive and seronegative individuals respectively. STATA 14 was also used to determine the relative risk of seropositivity for each risk factor. Analysed results were considered statistically significant if p ≤ 0.05.

## Results

In total, 300 participants were recruited for the study. Out of this number, 80.6% (n=242) were female, of which 45.5% (n=110) were pregnant. The median age for all participants was 27 years.

### Seroprevalence of *T. gondii*

**Figure 1.**
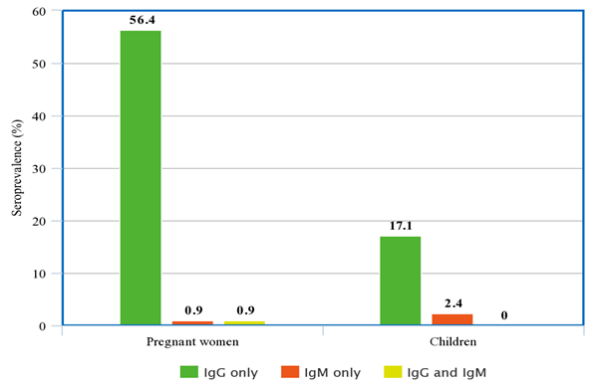
Seroprevalence of anti- *T. gondii* antibodies in children and pregnant women.

50.3% (n=151) of the 300 participants were seropositive for anti- *T. gondii* antibodies. Among the seropositive population, 98% (n=148) and 2% (n=3) of the participants were seropositive for IgG and IgM respectively, with 0.3% (n=1) of the participants seropositive for IgM but seronegative for IgG (IgM only), 49.3% (n=148) seropositive for IgG but seronegative for IgM (IgG only) and 1.3% (n=2) of the 151 seropositive participants seropositive for both IgG and IgM ( Table 1). Furthermore, 56.4% (n=62) of the pregnant women were seropositive for IgG only, with 0.9 % (n=1) testing positive for IgM only and both IgG and IgM. Out of the 41 children tested, 17.1% (n=7) were seropositive for IgG only whereas 2.4% (n=1) were seropositive for IgM only. None of the children were seropositive for both IgG and IgM ( Figure 1).

**Table 1.** Seroprevalence of anti- *T gondii* immunoglobulins.

Tests	Positive (n %)	Negative (n%)
IgG and/or IgM	151 (50.3)	149 (49.7)
IgM only	1 (0.3)	299 (99.7)
IgG only	148 (49.3)	152 (50.7)
IgG and IgM	2 (0.7)	298 (99.3)

 The communities within which the hospitals are located were classified as urban (Kumasi South Hospital), rural (Agona Government Hospital) and peri-urban (Kuntanase Government Hospital) based on their distance from Kumasi, the capital city. (Our classification is based on the population of the communities, where urban is highly populated city area, followed by peri-urban then, the rural area with the lowest population). Seroprevalence rates of 47.1%, 50.6% and 55.6% were therefore observed from the urban, rural and peri-urban areas, respectively ( Table 2a). Seroprevalence of anti- *T. gondii* IgG per study sites were 49.4% (n=39), 55.6% (n=45) and 47.1% (n=66) in the rural, peri-urban and urban areas respectively. Seropositivity for IgG and both IgG and IgM were 1.42% (n=2) in the urban area whereas seropositivity of 1.26% (n=1) for IgM was recorded in the rural area ( Table 2b).

**Table 2a.** Seroprevalence of *Toxoplasma gondii* based on the type of community.

Setting	Total tested	Seropositive (n %)	χ ^2^	P-value
			1.457	0.482
*Urban*	140	66 (47.1)		
*Peri-Urban*	81	45 (55.6)		
*Rural*	79	40 (50.6)		

**Table 2b.** Sero-prevalence of anti *T. gondii* IgG and IgM per study site location.

Location	IgG (n%)	IgM (n%)	IgG & IgM (n%)
**Urban**	66 (47.1)	2(1.42)	2 (1.42)
**Peri-Urban**	45 (55.6)	0	0
**Rural**	39 (49.4)	1(1.26)	0

A total of 110 pregnant women were recruited into the study, including 37 in their first trimester, 41 in the second trimester and 32 in the third trimester. The women ranged in age from 16 to 45 years, with a mean age of 28.6 years. Out of the 110 pregnant women, sixty-two were seropositive, indicating a general seroprevalence of 54.6%. Seropositivity for *T. gondii* was observed in 18 (48.6%) of the pregnant women in their first trimester, 22 (53.7%) in their second trimester and another 22 (68.8%) in their third trimester ( Table 3a). Almost all seropositive pregnant women within the first 16.4%(n=18), second 20% (n=22) and third 20% (n=22) trimesters were seropositive for only anti- *T. gondii* IgG. ( Table 3b).

**Table 3a.** Seroprevalence of anti- *Toxoplasma gondii* immunoglobulins in pregnant women.

	Total tested	Positive (n %)	Negative (n%)	χ2	RR (95% CI)	P-value
**Gestation period**				2.53	1.27(0.94-1.72)	0.1119
First trimester	37	18 (48.6)	19 (51.4)			
Second trimester	41	22 (53.7)	19 (46.3)			
Third trimester	32	22 (68.8)	10 (31.3)			

**Table 3b.** Sero-prevalence of anti *T. gondii* IgG and IgM among pregnant women.

Gestational Period	IgG (n%)	IgM (n%)	IgG & IgM (n%)
First trimester	18 (16.4)	0	0
Second trimester	22 (20)	0	0
Third trimester	22 (20)	1 (0.9)	1 (0.9)

To identify risk factors for *T. gondii* infection, the risk-ratio and chi-square tests were conducted to investigate the association between a few predisposing factors and seropositivity. Cat ownership (p=0.002) and contact with cat litter (p=0.003) were strongly associated with seropositivity for *T. gondii*. Handling of raw meat was also identified as a risk factor for exposure (p=0.05) ( Table 4).

**Table 4.** Association between *Toxoplasma gondii* seropositivity and possible risk factors.

Possible risk factors	Total tested	Positive n (%)	Negative n (%)	χ ^2^	RR (95%CI)	P-value
**Cat ownership**				10.10	1.76(1.23-2.53)	0.002**
Yes	92	59 (64.1)	33 (35.9)			
No	208	91 (43.8)	117 (56.3)			
**Contact with cat litter**				4.50	1.66(1.03-2.67)	0.003**
No	241	113 (46.9)	128 (53.1)			
Yes	59	37 (62.7)	22 (37.3)			
**Source of drinking water**				1.66	0.99(0.81-1.04)	0.198
Wells	71	40 (56.3)	31 (43.7)			
Pipe	223	107 (48.0)	116 (52.0)			
River	6	3 (50.0)	3 (50.0)			
**Handling of raw meat**				3.85	1.25(1.00-1.57)	0.050*
Yes	151	84 (55.6)	67 (44.4)			
No	149	66 (44.3)	83 (55.7)			
**Eating raw/undercooked** **vegetables**				1.98	1.05(0.98-1.14)	0.16
Yes	269	139(52.2)	130 (47.8)			
No	29	11 (33.3)	18 (66.7)			

Certain demographic factors were also investigated to ascertain their association with seropositivity. The results revealed that the location, sex, education and employment status of participants were not predisposing factors to seropositivity. Age was, however, significantly associated with seropositivity (p-value = 1.3*10 ^-6^) ( Table 5).

**Table 5.** Association between demographic factors and seropositivity of *Toxoplasma gondii*.

Demographics	Total tested	Positive (n %)	Negative (n%)	χ ^2^	RR (95% CI)	P-value
**Location**				1.07	1.12 (0.90-1.38)	0.3
Rural	81	45 (55.6)	36 (44.4)			
Urban	140	66 (47.1)	74 (52.9)			
Peri-Urban	79	40 (50.6)	39 (49.4)			
**Sex**				3.28	0.65 (0.40-1.04)	0.07
Male	58	23 (39.7)	35 (60.3)			
Female	242	127 (52.5)	115 (47.5)			
**Education**				0.95	0.96 (0.88-1.05)	0.33
None	40	23 (57.5)	17 (42.5)			
Basic	56	27 (48.2)	29 (51.8)			
JHS	84	51 (60.7)	33 (39.3)			
SHS	86	38 (44.2)	48 (55.8)			
Tertiary	30	10 (33.3)	20 (66.7)			
Adult education	4	2 (50.0)	2 (50.0)			
**Employment**				2.69	1.24 (0.96-1.6)	0.1
Unemployed	145	75 (51.7)	70 (48.3)			
Employed	152	75 (49.3)	77 (50.7)			
Retired	3	2 (66.7)	1 (33.3)			
**Age**				16.66	1.29 (1.14-1.46)	<0.001
0–18	67	19 (25.4)	56 (83.6)			
19–44	175	92 (52.7)	73 (41.7)			
45->	58	40 (67.0)	20 (34.5)			

### Effect of *T. gondii* infection on haematological parameters

*T. gondii* is capable of infecting virtually any nucleated cell, including erythroblasts. The effect of *T. gondii* on certain blood parameters (WBC, LYM, NEUT, RBC, Hb and MCV) was therefore investigated. Comparisons were made between seropositive and seronegative individuals for males and females. A further comparison was made between being pregnant or not for seropositive and seronegative females. The median and interquartile ranges were calculated, where p-values ≤ 0.05 were considered significant. We obtained significant differences in the WBC, LYM and MCV for males (p-values=0.0223, 0.0275, and 0.0271) respectively ( Table 6a) whereas for the females, significant association was obtained only in the MCV (p-value=0.0035) ( Table 6b). For the pregnant women who were seropositive, significant reduction was obtained in the LYM count (p=0.0369) ( Table 6c).

**Table 6a.** Effect of *Toxoplasma gondii* infection on haematological parameters in males

Haematological parameters	Seropositive	Seronegative	P-value
	Median (IQR)		
White blood cells (103/µL)	2.80 (2.60-3.30)	4.10 (2.60-4.80)	0.0223 *
Lymphocytes (103/µL)	1.90 (1.30-2.30)	2.30 (1.80-3.30)	0.0275 *
Neutrophils (103/µL)	1.00 (0.60-1.30)	1.10 (0.80-1.80)	0.2587
Red blood cell (106/µL)	4.64 (3.92-5.59)	4.99 (4.37-5.30)	0.6731
Haemoglobin (g/dl)	12.40 (11.70-14.60)	12.20 (11.00-13.90)	0.47
Mean corpuscular volume (/fL)	97.80 (94.20-102.90)	94.40 (87.90-100.00)	0.0271 *

* *P-values* ≤ *0.05*

**Table 6b.** Effect of *Toxoplasma gondii* infection on haematological parameters in females.

Haematological parameters	Seropositive	Seronegative	P-value
	Median (IQR)		
White blood cells (10 ^3^/µL)	3.20 (2.00-5.13)	3.45 (2.20-5.20)	0.3727
Lymphocytes (10 ^3^/µL)	1.50 (0.70-2.60)	1.55 (0.38-2.70)	0.955
Neutrophils (10 ^3^/µL)	0.70 (0.40-1.10)	0.80 (0.38-1.20)	0.7711
Red blood cell (10 ^6^/µL)	4.24 (3.75-4.56)	4.33 (3.94-4.64)	0.0825
Haemoglobin (g/dl)	11.35 (10.20-12.30)	11.40 (10.30-12.50)	0.531
Mean corpuscular volume (/fL)	100.10 (92.03-104.50)	96.40 (87.35-101.40)	0.0035 **

** *P-values* ≤ *0.01*

**Table 6c.** Effect of *Toxoplasma gondii* infection on haematological parameters in pregnant women.

Haematological parameters	Seropositive	Seronegative	P-value
	Median (IQR)		
White blood cells (10 ^3^/µL)	2.95 (1.80-5.95)	3.30 (1.65-5.88)	0.87
Lymphocytes (10 ^3^/µL)	1.00 (0.00-1.63)	0.00 (0.00-1.33)	0.0369 *
Neutrophils (10 ^3^/µL)	0.50 (0.00-0.80)	0.30 (0.00-0.80)	0.4237
Red blood cell (10 ^6^/µL)	4.21 (3.58-4.37)	4.06 (3.70-4.51)	0.6363
Haemoglobin (g/dl)	11.15 (10.20-11.90)	11.20 (10.25-12.73)	0.4645
Mean corpuscular volume (/fL)	98.00 (00.00-103.50)	92.40 (00.00-101.70)	0.139

* *P-values* ≤ *0.05*

## Discussion

*Toxoplasma gondii* is an intracellular parasite with a wide geographical distribution and an extremely broad host range. Despite the high prevalence of *T. gondii* infections in many parts of the world, there is limited data on the epidemiology of the infection in Ghana, and a majority of the few studies available are restricted to Accra, the capital city. Studies investigating the epidemiology of the infection typically do so by determining seroprevalence rates based on the detection of *T. gondii*-specific antibodies. *T. gondii*-specific IgG is associated with previous exposure to the parasite and is used as a marker for latent infection while IgM is used as a marker for exposure or acute infection ^16^.

In this study, an overall seroprevalence of 50.3% was observed, which is similar to a study from Accra that reported a seroprevalence rate of 49.7% ^17^, in contrast to another Accra-based study that reported 92.5% seroprevalence ^12^. The study also found a seroprevalence rate of 1.0% for parasite-specific IgM, suggesting active infections in these study participants. Other studies have previously reported fairly low seroprevalence rates of IgM, including 6.0% in the Central region of Ghana and 2.5% in Southwestern Ethiopia ^13, 18^. However, anti- *T. gondii* IgM seroprevalence rates as high as 29.7%, 39.1% and, even, 76.1% have been reported in Accra and Kumasi ^11, 12, 17^. Variations in the seroprevalence of anti- *T. gondii* antibodies from different studies may be due to methodological differences, including the choice of commercial ELISA kits.

As the three hospitals were located in different settlement types (urban, peri-urban and rural), we assumed that the patient population at each hospital primarily comprised individuals from the surrounding communities. We, therefore, compared seroprevalence rates among participants from the urban, peri-urban and rural communities. In many instances, rural inhabitants regularly come into contact with potentially oocyst-contaminated soil as part of their normal farming activities. However, we found no significant association between seropositivity and settlement type, which agrees with findings from Munoz-Zanzi *et al*., ^19^, who also found no obvious differences in seroprevalence between rural villages and urban slums. In contrast, Kawashima *et al*., ^20^ showed that seropositivity was significantly higher in rural communities compared to urban communities.

Acute infection by *T. gondii* in pregnancy can be detrimental to the foetus, resulting in stillbirth, chorioretinitis, hydrocephalus or even death ^21^. The ability of almost all subclasses of IgG — with the notable exception of IgG2 — to cross the placenta provides some form of protection to the foetus ^22^. The period of pregnancy in which the expectant mother becomes infected greatly influences the incidence and severity of the disease. Infection within the first trimester of pregnancy carries an approximately 6% risk of transmission to the foetus, while in the second and third trimesters, the risk of congenital transmission is about 33–47% and 60–81%, respectively ^21^. Contrary to the gestational risk of transmission, the risk of developing clinical symptoms of the disease (congenital toxoplasmosis) is highest when maternal infection occurs within the first trimester and gradually reduces through the second and third trimesters ^21^. Our study estimated an overall anti- *T. gondii* seroprevalence of 56.4% among pregnant women. This observation is lower than the 83.6% reported by Sefah-Boakye *et al*., in the same region (Ashanti) ^11^. Furthermore, we observed a trend of increasing seroprevalence with gestational period, with similar observations made in other studies from Kumasi, Accra and Southwestern Ethiopia ^11, 18, 23^.

In disease control, prevention and elimination, identification of the risk factors for infection are key. Horizontal transmission of *T. gondii* may occur via contact with cat litter, consumption of raw meat, eating of raw or undercooked vegetables, organ transplant and blood transfusion. We identified cat ownership, contact with cat litter and handling of raw meat as important risk factors for infection among our study population. Since *T. gondii* undergoes sexual reproduction in the gastrointestinal tract of domestic felines, individuals who owned cats or had regular contact with cat litter had 76% and 66% greater risk of *T. gondii* infection respectively compared to individuals who neither owned cats nor handled cat litter. Furthermore, since *T. gondii* is capable of encysting in the tissues of infected animals, people who regularly handle raw meat are also at greater risk of infection ^24^. We also investigated the association between certain demographic parameters and seropositivity. Consistent with findings from other studies in Ghana and Iran, seropositivity increased with age ^13, 15, 25^. This is due to the fact that infection has strongly been associated with contact with the soil where the oocyst prevails for years. The longer an individual survives, the greater the likelihood of coming into contact with contaminated soil hence the increase in seroprevalence with age.

*T. gondii* is capable of invading any nucleated cell, including immature red blood cells ^26^. As a mechanism of survival, intracellular parasites need to exit and re-infect other cells. The mechanism by which *T. gondii* exits infected cells is still not clearly understood. Some *in vitro* studies have demonstrated that *T. gondii* exits the cell by exerting tension on the host cell membrane, often causing the cell to rupture ^27^. Other studies have suggested that the parasite exits the host cell by disrupting the cytoskeleton in a manner similar to *Plasmodium*, resulting in cell lysis ^28^. In the previous report by Agordzo *et al.,* (2019), results obtained for seropositive and seronegative individuals were generally similar ^29^. This is not different from the present study where the haematological data had been expanded and comparisons made between similar groups. These observations agree with other findings where haematological parameters sometimes remain stable even in times of infection ^30^. With the exception of pregnant women, we found significant differences in the mean corpuscular volumes of *T. gondii* seropositive and seronegative males and females. The mean corpuscular volumes increased significantly in seropositive individuals for both males and females as compared to the seronegative groups. Furthermore, the mean corpuscular volume was reported to have reduced significantly in seropositive pregnant women in Libya which is not the case in our study ^30^. We observed an increase in the MCV of seropositive pregnant women, even though this was not significant. Consistent with other findings in Libya and Iraq, infection by *T. gondii* did not have any significant effect on the red blood cells count and haemoglobin level in pregnant women ^30, 31^. The WBCs are known to play a major role in protection against infection. Whiles the immune system is considered to be fighting infections in cases of elevated WBC count, a significant decrease can be considered to be detrimental to one’s health. Even though a general decrease in WBC counts was observed in all categories under consideration, a significant reduction was obtained for only seropositive males. Furthermore, our findings for the WBC count for pregnant women does not agree with the reports of Hassen *et al.,* (2019) which reported a significant decrease in the white blood cell for seropositive pregnant women ^30^. An investigation of the lymphocytes and neutrophils, which are components of the WBC and promote host defence by inducing anti-inflammatory response also reveal decreased counts in seropositive individuals for males and females whereas an increase in their counts were observed for pregnant women. The lymphocyte levels in seropositive males reduced significantly as opposed to the seronegative groups. In the case of the pregnant women, the lymphocyte count increased significantly in the seropositive group and this conforms with the findings of Hassen *et al.,* (2019), even though they did not report a significant observation ^30^. Per the experimental observations of Biswas *et al.,* (2017) ^32^, depletion of neutrophils results in a reduction in the IFN-γ production which resulted in an increase parasite burden in the central nervous system. While the exact molecular processes driving these differences are still unclear, there is increasing evidence that latent toxoplasmosis may cause chronic low-grade inflammation ^33^, which may, in turn, lead to anaemia ^34^.

## Conclusion

The seroprevalence of *T. gondii* infection was high among our study population, including among pregnant participants. In addition, cat ownership, contact with cat litter and age were identified as major risk factors for infection. Furthermore, additional research is needed to fully clarify the links between latent toxoplasmosis and anaemia. In conclusion, we recommend that testing for infection by the parasite be included in routine screening of pregnant women seeking antenatal care and further studies should investigate the impact of infection on blood parameters of humans.

## Data availability

### Underlying data

Harvard Dataverse: Replication Data for: Seroprevalence, Risk Factors and Impact of Toxoplasma gondii Infection on Haematological Parameters in the Ashanti Region of Ghana: A Cross-Sectional Study, https://doi.org/10.7910/DVN/HXJONT ^35^.

This project contains the following underlying data:

- Data includes, demography of study participants, risk factors, IgG response and measurement of haematological parameters
- Data dictionary

### Extended data

Harvard Dataverse: Replication Data for: Seroprevalence, Risk Factors and Impact of Toxoplasma gondii Infection on Haematological Parameters in the Ashanti Region of Ghana: A Cross-Sectional Study, https://doi.org/10.7910/DVN/HXJONT ^35^.

This project contains the following extended data:

- Questionnaire used in the present study.

Data are available under the terms of the Creative Commons Zero "No rights reserved" data waiver (CC0 1.0 Public domain dedication).
