# Supplementary material for: Seroprevalence, risk factors and impact of Toxoplasma gondii infection on haematological parameters in the Ashanti region of Ghana: a cross-sectional study
Source: AAS Open Res. 2020 Jun 17;2:166. Originally published 2019 Nov 26. [Version 2] doi: 10.12688/aasopenres.13022.2 (PMC7369427; doi:10.12688/aasopenres.13022.2)
Supplement: Peer review history

# Peer review: Reviewer response for version 2

**DOI:** 10.21956/aasopenres.14190.r27496

**Published:** 2020-07-17

**Authors:** Rasool Jafari

**Affiliations:** 1 Department of Medical Parasitology, School of Medicine, Urmia University of Medical Sciences, Urmia, Iran

The authors have made some improvements in the manuscript, and now it can be accepted.

Is the work clearly and accurately presented and does it cite the current literature?

Partly

If applicable, is the statistical analysis and its interpretation appropriate?

Yes

Are all the source data underlying the results available to ensure full reproducibility?

Yes

Is the study design appropriate and is the work technically sound?

Partly

Are the conclusions drawn adequately supported by the results?

Yes

Are sufficient details of methods and analysis provided to allow replication by others?

Yes

Reviewer Expertise:

Molecular and serological epidemiology of parasitic diseases (e.g., toxoplasmosis, cryptosporidiosis and hydatid disease)

I confirm that I have read this submission and believe that I have an appropriate level of expertise to confirm that it is of an acceptable scientific standard.

# Peer review: Reviewer response for version 2

**DOI:** 10.21956/aasopenres.14190.r27497

**Published:** 2020-06-24

**Authors:** Emmanuel Awusah Blay

**Affiliations:** 1 Department of Pathology and Microbiology, University of Nebraska Medical Center, Omaha, NE, USA

The updated manuscript largely satisfies all concerns raised.

Is the work clearly and accurately presented and does it cite the current literature?

Yes

If applicable, is the statistical analysis and its interpretation appropriate?

Yes

Are all the source data underlying the results available to ensure full reproducibility?

Yes

Is the study design appropriate and is the work technically sound?

Yes

Are the conclusions drawn adequately supported by the results?

Partly

Are sufficient details of methods and analysis provided to allow replication by others?

Yes

Reviewer Expertise:

Molecular Parasitology and Microbial genetics

I confirm that I have read this submission and believe that I have an appropriate level of expertise to confirm that it is of an acceptable scientific standard.

# Peer review: Reviewer response for version 1

**DOI:** 10.21956/aasopenres.14110.r27426

**Published:** 2020-05-04

**Authors:** Emmanuel Awusah Blay

**Affiliations:** 1 Department of Pathology and Microbiology, University of Nebraska Medical Center, Omaha, NE, USA

The manuscript titled “Seroprevalence, risk factors and impact of *Toxoplasma gondii* infection on haematological parameters in the Ashanti region of Ghana: a cross sectional study” by Agordzo et al., presented evidence on the current serological prevalence of *T. gondii* and association with selected haematological parameters.

 Though the seroprevalence of *T. gondii* has been widely published in different risk groups in Ghana, this research adds to the body of existing literature and knowledge and presents evidence on the possible association between *T. gondii* infections and haematological parameters. Additionally, this research provides additional evidence on possible regional variations in seroprevalence in Ghana, as most of the exixting research has been conducted on the coastal regions of the country. Different geographical regions may possibly present additional evidence of variations in infections and associated risk patterns of *T. gondii.*

 Comments:

1. In providing the data for the seroprevalence for the study in Table 1, the authors provided the general seroprevalence from the study. However, considering that the data was obtained from different populations i.e. Pregnant women, infants, critically ill persons and general hospital attendees, it will be more informative to show which of these groups showed higher prevalence in IgG only, IgM only and both IgG and IgM. Such details would provide details as to which groups are most susceptible to T. gondii infections.
2. In Table 2, do the authors group seropositive individuals together in the different geographical settings. However, a breakdown on the type of seropositivity i.e. IgG only, IgM only and both IgG and IgM may be more beneficial to the readers for a critical understanding as to which geographical setting is recording more acute cases and which is showing more in exposure to *T. gondii*
3. In Table 3, the authors provide details of seropositivity in pregnant women. In total, 110 pregnant women comprising of 37 in first trimester, 41 in second trimester and 32 in third trimester are presented. Although this information may have a degree of importance, the gestation period alone is scanty to make any meaningful deductions. It will be more beneficial if the authors can add the gravidity and parity status of the pregnant women to show whether they have had previous live births or abortions. This additional information coupled with the specific seropositivity i.e. IgG only, IgM only and both IgG and IgM will provide information to discuss the significance of past or acute infections have on seropositivity.
4. In Table 5, the authors provide data on the haematological parameters analyzed and the seropositivity of the participants. Although this seems to be the crux and novelty of the paper, the manner the data has been presented provides the reader with less clarity. Because the participants were drawn from different population groups, it will be more prudent to provide a breakdown of the haematological data in sub-populations. This is so because the haematological parameters of pregnant women will be significantly different from children and also different from general hospital attendees. Thus, whiles providing a composite data is good, it does little to clearly draw out the differences between the various study groups. Additionally, I will suggest that the seopositivity data is clearly distinguished between IgG only, IgM only and both IgG and IgM. This provides greater clarity and brings out the different nuances in the data set.
5. The authors provide no data on the risk factors in this manuscript although it is an integral part in their title. I suggest they add the data or modify their title to reflect the data that is presented.

Minor Comments:

1. In paragraph 4, of sub-title “Seroprevalence of T. gondii” the authors state discuss results pertaining to risk factors and incorrectly attribute it to Table 3.
2. On the haematological data discussion, the authors should discuss the differences in mean corpuscular volumes. I am highly convinced that the differences observed could be due to large percentage of the participants being pregnant women.
3. A major limitation to this study is the use of a qualitative commercial kit for
  determining seropositivity. An additional method such as an avidity ELISA or molecular identification of specific genes of *T. gondii* such as the Dense granules (GRA) or surface antigens (SAG) may have provided more information whether the infections were latent or acute. I suggest the authors add the limitations of this study into their conclusions.

Is the work clearly and accurately presented and does it cite the current literature?

Yes

If applicable, is the statistical analysis and its interpretation appropriate?

Yes

Are all the source data underlying the results available to ensure full reproducibility?

Yes

Is the study design appropriate and is the work technically sound?

Yes

Are the conclusions drawn adequately supported by the results?

Partly

Are sufficient details of methods and analysis provided to allow replication by others?

Yes

Reviewer Expertise:

Molecular Parasitology and Microbial genetics

I confirm that I have read this submission and believe that I have an appropriate level of expertise to confirm that it is of an acceptable scientific standard, however I have significant reservations, as outlined above.

# Peer review: Reviewer response for version 1

**DOI:** 10.21956/aasopenres.14110.r27328

**Published:** 2020-01-14

**Authors:** Rasool Jafari

**Affiliations:** 1 Department of Medical Parasitology, School of Medicine, Urmia University of Medical Sciences, Urmia, Iran

I studied the manuscript with interest. The manuscript studied the prevalence and related risk factors of infection with *T. gondii* in Ghana. There are some issued that need to be addressed as below.

1. There is extensive research on the seroprevalence of toxoplasmosis, and most of the risk factors and epidemiological aspects of the infection are relatively known. The findings of the manuscript are not so novel, mostly known; however it has regional importance in the studied area of Ghana.
2. The method for determining anti- *Toxoplasma* IgG and IgM is not quantitive, and I do not suggest rapid tests for research. If the author may have used ELISA, ECLA or CLA, data may have been more accurate and they may analyze the quantitative concentration of antibodies among risk factors.
3. For studying the hematological parameters, authors should be aware of the fact that the study population must be similar in seropositive and seronegative individuals. In women, anemia is prevalent and the authors could not compare the parameters between men and women; they may analyze it separately among both sexes. Pregnancy and nutritional status during pregnancy (food, vitamin and iron supplements) are other issues affecting blood parameters, that are not addressed, which can bias the results.
4. The authors could discuss other risk factors reported in the literature, such as having contact with soil and food habits such as eating raw vegetables, etc.
5. Why are different mean corpuscular volumes among seronegative and seropositive cases? The authors should discuss this issue and I think it may reflect the fact that the majority of the studied group were female and pregnant.
6. The language is relatively good; however, few misspellings and punctuation-errors need to be corrected.

Is the work clearly and accurately presented and does it cite the current literature?

Partly

If applicable, is the statistical analysis and its interpretation appropriate?

Yes

Are all the source data underlying the results available to ensure full reproducibility?

Yes

Is the study design appropriate and is the work technically sound?

Partly

Are the conclusions drawn adequately supported by the results?

Yes

Are sufficient details of methods and analysis provided to allow replication by others?

Yes

Reviewer Expertise:

Molecular and serological epidemiology of parasitic diseases (e.g., toxoplasmosis, cryptosporidiosis and hydatid disease)

I confirm that I have read this submission and believe that I have an appropriate level of expertise to confirm that it is of an acceptable scientific standard, however I have significant reservations, as outlined above.
